# Asymmetric Adaption in Social Learning: Understanding the Dilemma of Competition and Cooperation

**DOI:** 10.3390/bs14080721

**Published:** 2024-08-16

**Authors:** Siying Li, Yulong Huang, Cheng Xu, Jie Wu, Chen Qu

**Affiliations:** 1 Faculty of Education, Northeast Normal University, Changchun 130024, China; lisiying@nenu.edu.cn; 2 Department of Experimental Psychology, Ghent University, 9000 Ghent, Belgium; yulong.huang@ugent.be; 3 Key Laboratory of Brain, Cognition and Education Sciences, Ministry of Education, School of Psychology, Center for Studies of Psychological Application, Guangdong Key Laboratory of Mental Health and Cognitive Science, South China Normal University, Guangzhou 510631, China; 2016022547@m.scnu.edu.cn; 4 School of Psychology, Fujian Normal University, Fuzhou 350117, China; wuj@fjnu.edu.cn

**Keywords:** competition, cooperation, chicken game, social learning, social dilemma

## Abstract

The constant challenge in social interactions involves making informed decisions in the face of competitive and cooperative dilemmas. The decision-making process can be influenced by various factors present in the social context. According to the behavior-pattern-categorization framework of information acquisition, potential biases may develop at all stages of decision-making as information about social context is progressively entered and integrated. In this study, employing the Chicken Game, we investigated the influence of varying information levels within the behavior-pattern-categorization framework (i.e., competitiveness of behavior choice, uncertainty of behavior pattern, and sociality of category) on decision-making in the dilemma of competition and cooperation. Combined with reinforcement learning models, our findings from three experiments showed that participants exhibited basic complementary behavior, becoming less competitive against highly competitive opponents and vice versa. Notably, individuals exhibited varying adaptation rates to different levels of opponent competitiveness and fluctuations. Specifically, participants adapted slower to highly competitive opponents and faster to cooperative opponents. This asymmetric adaptation in social learning is related to the rate at which various levels of information are updated. The current study disentangles the different levels of information acquisition and highlights the asymmetric processing that can occur during the updating of information within each level.

## 1. Introduction

‘To compete or cooperate’ presents a classic social dilemma that challenges human sagacity: excessive dominance can lead to mutual detriment, while unconditional compromise may be vulnerable to exploitation. This dilemma is prevalent among individuals, groups, and even nations, impacting personal well-being and development, as well as societal stability and prosperity [1,2,3,4,5]. In such dilemmas, making any sudden moves before obtaining adequate information about the others’ traits is unwise. The behavior and traits of others serve as benchmarks to inform our own actions [6,7,8]. We process such external information through social learning, transforming it into internalized knowledge, which subsequently is interpreted and influences our decision-making. However, this process can be biased [9,10]. Utilizing a social dilemma modeled through chicken games, the current study aims (i) to investigate how individuals’ behavioral choices between competition and cooperation are influenced by their partners’ competitiveness, fluctuation, and social categorizations and (ii) to examine the underlying cognitive mechanisms of information processing and updating within the reinforcement learning framework.

The competition–cooperation dilemma intertwines the interests of individuals and groups, yielding profound implications for society. To understand this complex phenomenon, researchers have devised simple yet elegant games that delve into the core aspects of social dilemmas, such as Prisoner’s Dilemma and Chicken Game [5,11]. These games capture core elements of social dilemmas in real life and provide much experimental evidence for theory development. The interdependence theory in game theory serves as an integrative framework for analyzing social interactions and interpersonal relationships. This theory categorized interdependent interactions as a combined function of the structure (e.g., the score matrix and game rules), the interacting partners, and interaction dynamics [5,12]. Within this framework, previous research has mainly focused on the first two static factors such as the game’s structure and context [13,14,15], players’ social categorization [16,17], personality traits [4,18], and hormones [19,20]. Some dynamic factors in interpersonal interactions, such as strategy and communication, have been gradually explored [1,21].

However, one often overlooked dynamic aspect is the acquisition of social information, which evolves over time as players observe their opponents’ behaviors. Learning about an opponent’s behavior patterns through their binary choices, whether competitive or cooperative, is a gradual process that shapes decision-making. More competition choices indicate a higher competitive nature of the opponent and vice versa. This information learning occurs step by step, highlighting the dynamic nature of cognitive processing. Understanding these behavior patterns is crucial for strategic planning, but the continuous update of this information poses challenges for individuals to grasp the complete picture all at once, increasing space for subjective interpretation. Thus, a fundamental question is raised: how do the characteristics and choices of opponents shape our learning process and decision-making within a competitive–cooperative dilemma?

Social behaviors and characteristics of others play a crucial role in influencing decision-making processes, often leading to observable differences in behaviors, preferences, or biases. These behavioral performances often do not arise independently [22,23]. Social learning is a complex, multi-layered process where individuals acquire information from their social environment, helping them make quick and rational decisions [24,25]. This dynamic process involves assimilating different types of information: (i) primary information, which is directly observed or acquired from the social context, such as behaviors or feedbacks; (ii) secondary information, consisting of behavioral patterns or certain characteristics of others that become more apparent as primary information accumulates; and (iii) more abstract tertiary information known as social categorizations (i.e., labeling), which can be described and generalized based on prior experiences or beliefs (e.g., perceiving friends as more cooperative) [14,26,27]. Each level of information can influence our decision-making, and biases may occur at every stage of information processing. The inherent challenges include the incomplete nature of primary information, the varied interpretations of secondary information, and the abstractness of tertiary information, posing obstacles to forming an objective understanding of the social world.

Studies have demonstrated that primary information, like the binary feedback of victory versus defeat or gain versus loss, has different effects or values for individuals. One study demonstrated that coding for victories and defeats is located in different brain regions; specifically, the ventromedial prefrontal cortex and ventral striatum react to competitive victories and defeats [28]. On the secondary information level, research shows that moral judgments are subject to asymmetric Bayesian updating, with higher volatility in beliefs about bad agents compared to good agents [29], indicating a bias in updating behavioral patterns. In terms of tertiary information, its impact on individuals’ decision-making is profound. A study outlined the process of parochial cooperation, where individuals rapidly categorize others as in-group or out-group, forming initial cooperative preferences, which are then modified based on interaction outcomes [9]. These findings underscore the necessity of unraveling the influences of different information levels on behavioral decisions within dynamic social learning, to achieve a deeper understanding of the integral interplay of behavior-pattern-categorization framework.

Our study delves into social learning processes within competition–cooperation dilemmas through three experiments. We utilized reinforcement learning models to decode and understand the intricate cognitive mechanisms underpinning social learning and decision-making to disentangle the complex interactions between behavior, pattern, and categorization. In Experiment 1, participants engaged in the Chicken Game against opponents characterized by high or low levels of competitiveness, assessing the effect of opponent behavior and behavioral patterns on the participants’ social learning and decision-making processes. In Experiment 2, we introduced an additional layer of complexity by incorporating tertiary information, where participants contended against either human or computer opponents, each with distinct competitive levels, exploring the impact of this expanded information on decision-making in competitive–cooperative dilemmas. In Experiment 3, we designed an uncertain scenario, presenting participants with a range of human and computer opponents whose behavioral patterns were fluctuated and unpredictable. By introducing such complexity, we sought to elucidate how different information levels, when intertwined, influence decision-making in dynamically competitive environments. Overall, by integrating the Chicken Game with reinforcement learning models across these three experimental setups, the study aims to unravel the influence of varying information levels on social learning and decision-making in the dilemma of competition and cooperation.

## 2. Experiment

This research was reviewed and approved by the Ethical Committee of the Department of Psychology at South China Normal University (SCNU-PSY-2021-234). Informed consent was obtained from all participants before the formal experiments. Across all experiments, sample sizes were determined in advance to ensure sufficient power to detect small-to-medium effect sizes. To ensure the robustness of our findings, we employed both frequentist and Bayesian statistical methods. Finally, all participants were fully debriefed at the end of the experiment, where they were informed about the study’s purpose and the manipulation of the opponents and competition conditions.

### 2.1. Experiment 1

In Experiment 1, within the context of the Chicken Game, we designed a competition and cooperation dilemma that incorporated two distinct types of information: the individual decisions made by their opponent in each trial and the overarching behavioral pattern displayed by the opponent throughout the game. In this experiment, we aimed to investigate how these two forms of information—the immediate choices and the broader behavioral tendencies of the opponent—impact the decision-making strategies of the participants.

#### 2.1.1. Methods

- Participants

A priori sample size estimation was conducted using G. Power V3.1 with the following parameters: repeated-measure design factors = 2 × 5; effect size = 0.25; significance level = 0.05; and power = 0.9. The power analysis resulted in a sample of twenty-six participants. Twenty-nine participants (aged 19.38 ± 1.68 years, 17 females) were recruited via internet advertisements and provided informed written consent. All participants are right-handed, with no history of psychiatric or neurological disorder, and have normal or corrected normal vision.

- Experimental design and procedure

**Figure 1:**
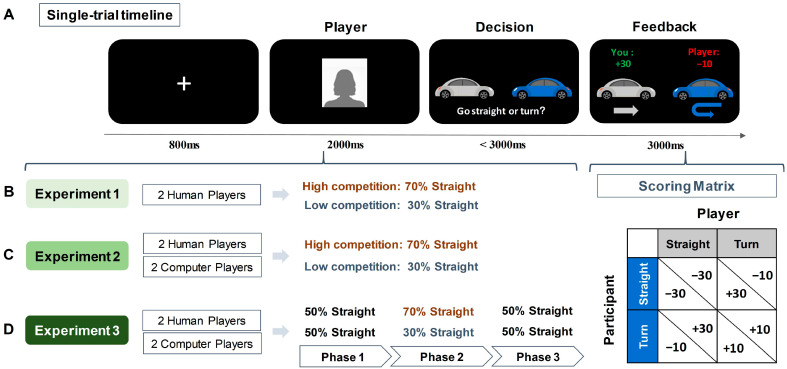
Illustration of the experimental task and designs. (**A**) Single-trial timeline and scoring matrix of the Chicken Game. (**B**) Design of Experiment 1. Participants were told they were playing against two human opponents, represented by human faces (a silhouette is used here for illustration). Unbeknownst to participants, opponents had preprogrammed competition tendencies—one highly competitive opponent (70% chance to go straight) and one less competitive opponent (30% chance to go straight). Participants had to learn opponents’ competition rates through the trial feedback. (**C**) Design of Experiment 2. Participants interacted with four opponents, two identified as humans and the other two as computers. Unbeknownst to participants, both humans and computers were preprogrammed and included one highly competitive opponent and one less competitive opponent. (**D**) Design of Experiment 3. Similar to Experiment 2, with participants encountering both human and computer opponents, but with their preprogrammed competitiveness shifting across three phases.

Participants were introduced to engage in the Chicken Game, where they were asked to imagine they were driving towards an opponent at an intersection, with the choice to go straight (indicating competition) or to turn (indicating cooperation). The scoring rule was as follows (see Figure 1 for scoring matrix): if both players chose to turn, each received 10 points; if one player chose to go straight while the other turned, the straight-going player received 30 points and the turning player lost 10 points; and if both players chose to go straight, each lost 30 points. Participants were aware that the outcome of each trial was contingent on the decisions of both players. During the game, they encountered two opponents, presented as former participants but actually simulated. They were told that they were playing against opponents who had previously participated in the experiment and that their past decisions were being used in the current game. However, in reality, these opponents were simulated and preprogrammed. This approach was used to create more realistic and socially engaging interactions, thereby capturing more genuine responses and enhancing the ecological validations. One opponent had a high competitive tendency (70% chance of going straight) and the other had a low competitive tendency (30% chance of going straight).

As illustrated in Figure 1, each trial began with an 800 ms fixation, followed by a 2000 ms presentation of the opponent’s picture. The pictures of human opponents were selected from our laboratory’s custom database and all pictures were of university students with neutral facial expressions. In each trial, participants saw pictures of human faces to indicate which player they were playing within the current trial. The decision screen showed a gray car (representing the participant) and a blue car (representing the opponent). Participants were instructed to press either the ‘f’ key to go straight or the ‘j’ key to turn within 3000 ms. The key assignments and the car representations were counterbalanced among participants. Non-responsive trials within 3000 ms were excluded from subsequent analysis. Following a choice, the outcome of the current trial was presented for 3000 ms. The game comprised 200 trials over 5 blocks. Within each block of 40 trials, there were 20 trials against a highly competitive opponent and 20 trials with a lower-competitive opponent, randomly presented. Prior to the formal experiment, participants underwent 4 practice trials, which were identical to the formal experiment, but with labels ‘name 1’ and ‘name 2’ instead of opponent pictures, to familiarize themselves with the task without prior knowledge of the opponents.

- Model construction and estimation

To investigate the potential link between internal cognitive mechanisms and behaviors in the competition–cooperation dilemma, we employed a computational modeling approach within a repeated Chicken Game. Specifically drawing upon the reinforcement learning framework and incorporating the behavior-pattern-categorization information framework, we constructed three computational models. In this experiment, we considered two types of information: primary information (e.g., behavior choices) and secondary information (e.g., behavior patterns). The reinforcement learning-based models integrated two key aspects: the decision-making process, where participants aimed to maximize their benefits, and the updating process, which aimed to minimize the disparity between actual outcomes and expectations using the Rescorla–Wagner prediction error rule. This allowed participants to learn about the competition level of their opponents and adapt their strategies to maximize potential rewards. For example (Model_1B), we defined the expectation of the probability of an opponent choosing competition in the current trial (*P*_(*t*+1)_, Equation (1)) as a function of the current expectations (*P*_(*t*)_) and the discrepancy between these expectations and the actual feedback experienced (*γ*_(*t*)_, where *γ* = 1 when the partner chooses competition, and *γ* = 0 when the partner chooses cooperation behavior). This discrepancy is known as the prediction error (*PE*_(*t*)_, Equation (2)), multiplied by a learning rate (α) where *B* indexes different opponent’s behavioral choices [30]. Learning rate is an important free parameter in reinforcement learning models which restricts and scales how much the degree of prediction error will be taken into internal computation [30]. As learning rates vary among participants, they reflect the extent to which individuals learn through prediction errors. The competition rate is subsequently transformed into an expected value when participants choose competition behavior (Equation (3)) or cooperation behavior (Equation (4)). Following this, the expected value (*EV_t_*) was calculated using a softmax function to determine the probability of a participant’s choice with a given partner (*IP*, Equation (5)). The parameter *β* in the softmax function reflects whether a participant is more likely to behave in a more explorative or exploitative manner.

In this study, we constructed distinct computational models, each tailored to analyze the impact of different levels of information processing—behavior, pattern, and categorization—both independently and in conjunction. Experiment 1 constructed three different models which varied in their assignment of the learning rate (α). In Model_1B, we assigned different α values for opponents’ choices of competition (α_B1_) or cooperation (α_B2_). This allowed us to explore the individual effect of primary information (e.g., the behavior of the opponents). Model_1P was developed to investigate the independent influence of behavior patterns, assuming varying learning rates for different competitive patterns (α_P1_ for the highly competitive opponent and α_P2_ for the less competitive opponent). In contrast, Model_1BP aimed to explore the combined impact of behavior and pattern by setting distinct learning rates for different behaviors and patterns (α_B1,P1_ for the highly competitive opponent’s choice to compete; α_B1,P2_ for the less competitive opponent’s choice to compete; α_B2,P1_ for the highly competitive opponent’s choice to cooperate; and α_B2,P2_ for the less competitive opponent’s choice to cooperate). This allowed us to explore how the combination of primary information (behavior) and secondary information (pattern) influences our decision-making (see Supplementary Information for more detail).

The parameters of these models were estimated by log-likelihood estimation using the maximizing function in Equation (6), where *n* indicates the total number of trials. To select a more representative model among the three, we used the Akaike Information Criterion (AIC) [31], which applies a penalty scaled by the number of free parameters of a complicated model. These estimations and model comparisons were carried out using custom MATLAB scripts.

Model_1B
  (1) $${P(d)}_{t+1}={P(d)}_{t}+\alpha_{B}*{PE}_{t}$$
  (2) $${PE}_{t}=\gamma_{t}-{P(d)}_{t}$$
  (3) $${EV(d)}_{t}={P(d)}_{t}*\left(-30\right)+(1-{P\left(d\right)}_{t})*30$$
  (4) $${EV(s)}_{t}={P(d)}_{t}*\left(-10\right)+(1-{P\left(d\right)}_{t})*10$$
  (5) $${IP(d)}_{t}=\frac{e^{\frac{{EV(d)}_{t}}{\beta }}}{e^{\frac{{EV(d)}_{t}}{\beta }}+e^{\frac{{EV\left(s\right)}_{t}}{\beta }}}$$
  (6) $$LLE=\sum_{t=1}^{n}\log({IP}_{t})$$

#### 2.1.2. Statistical Analysis

The behavioral data and model-derived estimated parameters were analyzed using JASP V0.16.4 [32]. To identify statistical significances, a Fisherian statistical approach was employed, setting the significance level at *p* < 0.05 after the Bonferroni correction was adopted. Repeated-measures analyses of variance (RM ANOVAs) were applied to investigate differences between the conditions. The effect sizes for the main effects and interactions in the ANOVAs were quantified using partial eta-squared (*η_p_*^2^*),* Cohen’s *d*, and 95% confidence intervals (CIs). Additionally, a Bayesian approach was adopted to evaluate the experimental hypothesis against the null hypothesis, using the Bayes factor (BF10) as a measure of the probability associated with the alternative hypothesis (H1) over the null hypothesis (H0) [33]. The statistical analysis protocol is identical to all experiments in our study.

#### 2.1.3. Result

- Behavioral results

**Figure 2:**
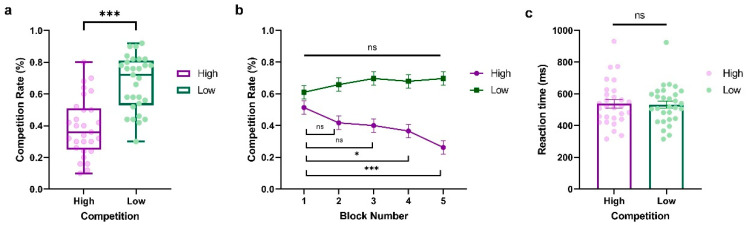
Results from Experiment 1. (**a**) Average percentage of decisions to competition (go straight), (**b**) block-to-block evolution of competition rate, and (**c**) average reaction time (ms) when participants play against highly and less competitive opponents. The *y*-axis indicates the raw performance data, with each dot representing an individual participant (* indicates *p* < 0.05, *** indicates *p* < 0.001, and ns indicates non-significant; error bars represent SEM).

To investigate the dynamics of competition behavior in response to varying opponent competition levels over time, we conducted a two-way repeated-measure ANOVA of the competition rate with Competition (High vs. Low) and Block (Block1~Block5) as within-subject factors, where Block represents the behavioral dynamic evolving across time. As shown in Figure 2a, we observed a significant main effect of Competition (*F*_(1,28)_ = 43.54, *p* < 0.001, *η_p_*^2^ = 0.609, BF_10_ = 2.748 × 10^8^), indicating that participants exhibited higher competitive behaviors when against a less competitive player (LP) compared to a highly competitive player (HP) (HP: *M* = 0.392, *SD* = 0.184; LP: *M* = 0.668, *SD* = 0.172). The main effect of Block was not significant (*F*_(4,114)_ = 2.377, *p* = 0.056, *η_p_*^2^ = 0.078, BF_10_ = 7264.794). Interestingly, we found a significant interaction between Competition and Block (*F*_(4,112)_ = 8.64, *p* < 0.001, *η_p_*^2^ = 0.229, BF_10_ = 36410.563, see Figure 2b), which demonstrated that the competitive behaviors of participants fluctuated over time based on the competition condition. This trend was particularly notable against highly competitive players, where participants showed a significant progressive decrease in competitiveness (Block 1 vs. Block 2: *t* = 2.301, *p* = 1.000, Cohen’s *d* = 0.423, 95% CI = [−0.218, 1.064]; Block 1 vs. Block 3: *t* = 2.712, *p* = 0.324, Cohen’s *d* = 0.498, 95% CI = [−0.153, 1.150]; Block 1 vs. Block 4: *t* = 3.534, *p* = 0.022, Cohen’s *d* = 0.649, 95% CI = [−0.028, 1.327]; Block 1 vs. Block 5: *t* = 6.000, *p* < 0.001, Cohen’s *d* = 1.103, 95% CI = [0.317, 1.888]). In contrast, participants showed stable and slightly more competitive behaviors when they were against an LP, with no significant difference among the five blocks (All *p*s > 0.05). Furthermore, we also conducted a paired-sample t-test on reaction time with Competition (High vs. Low) as a within-subject variable. No significant effect was found (*t_(_*_28)_ = 0.397, *p* = 0.695, Cohen’s *d* = 0.074, 95% CI = [−0.291, 0.437], BF_10_ = 44,285.091; HP: *M* = 536.938, *SD* = 138.061; LP: *M* = 531.690, *SD* = 121.492, see Figure 2c).

- Revelation in the computational model

The comparative analysis of the computational models revealed that Model_1B was more effective in fitting the participants’ behaviors than Model_1P (*t*_(28)_ = −3.34, *p* < 0.01, Cohen’s *d* = −0.621) and Model_1BP (*t*_(28)_ = −12.45, *p* < 0.001, Cohen’s *d* = −2.312). Based on Model_1B, a paired-sample *t*-test showed that participants had higher learning rates in response to cooperative choices by others than competitive choices (*t*_(28)_ = −2.31, *p* = 0.028, Cohen’s *d* = −0.430, 95% CI = [−0.807, −0.045], BF_10_ = 1.920; HP: *M* = 0.058, *SD* = 0.061; LP: *M* = 0.212, *SD* = 0.359). Detailed results of the model estimation and comparison are shown in Table 1.

### 2.2. Experiment 2

In Experiment 2, following a similar setup to Experiment 1, we extended the range of information in the context of the Chicken Game. In this experiment, we introduced a new layer of categorization, where participants encountered opponents who were either human or computerized opponents, each exhibiting either high or low competitive behaviors. We aimed to investigate how the categorization information involving the dynamic among behavior, pattern, and categorization impacts our decision-making strategies.

#### 2.2.1. Methods

- Participants

A priori sample size estimation was conducted using G. Power V3.1 with the following parameters: repeated-measure design factors = 2 × 2 × 5; effect size = 0.25; significance level = 0.05; and power = 0.9. The power analysis resulted in a sample of twenty-eight participants. An independent group of thirty participants (aged 20.86 ± 1.10 years, 17 females) were recruited through internet advertisements and provided informed written consent. Data from one participant were excluded from the final analysis due to a high number of non-responses trials. Thus, the final analysis sample size was twenty-night participants. All participants are right-handed, with no history of psychiatric or neurological disorder, and have normal or corrected normal vision.

- Experimental design and procedure

In Experiment 2, participants were engaged in the Chicken Game, where they were instructed to imagine a scenario of driving towards another car at an intersection with the choice to go straight or turn. The scoring rules and trial structure were identical to Experiment 1 (see Figure 1). However, in Experiment 2, participants encountered four opponents: two human players (social) and two computer players (non-social), each categorized as either highly competitive (70% chance of going straight) or less competitive (30% chance of going straight). Similar to Experiment 1, during trials involving human opponents, participants saw pictures of human faces, whereas trials with computer opponents displayed distinct computer images.

The task consisted of 5 blocks, totaling 200 trials, with each block comprising an equal number of trials against each of the four opponent types, randomly presented: highly competitive human player (HHP), less competitive human player (LHP), highly competitive computer player (HCP), and less competitive computer player (LCP). Prior to the formal experiment, participants underwent 4 practice trials where instead of pictures, opponents were identified by labels such as ‘name 1’, ‘name 2’, ‘computer 1’, and ‘computer 2’.

- Model construction and estimation

To investigate the effects of behavior, pattern, and categorization on social learning in Experiment 2, we constructed seven reinforcement learning models. Model_2B explored the influence of primary information, focusing on whether participants have different learning rates for different behavioral choices (α_B1_ for competition and α_B1_ for cooperation) of the opponent. Model_2P investigated secondary information to determine whether participants have different learning rates for opponents with different behavioral patterns (α_P1_ for the highly competitive opponent and α_P2_ for the less competitive opponent). Model_2C verified the effect of tertiary information and examined whether participants process opponents of different categorizations (social or non-social) using different learning rates (α_C1_ for the human opponent and α_C2_ for the computer opponent). Model_2BP examined the joint influence of behavior and pattern, setting different learning rates for different behaviors and patterns (α_B1,P1_ for the highly competitive opponent’s choice to compete; α_B1,P2_ for the less competitive opponent’s choice to compete; α_B2,P1_ for the highly competitive opponent’s choice to cooperate; and α_B2,P2_ for the less competitive opponent’s choice to cooperate). Model_PC tested the joint influence of behavior and categorization, setting different learning rates for different patterns and categorizations (α_P1,C1_ for the highly competitive human opponent; α_P1,C2_ for the highly competitive computer opponent; α_P2,C1_ for the less competitive human opponent; and α_P2,C2_ for the less competitive computer opponent). Model_2BC explored the possibility of joint influences of behavior and categorization on decision-making, using different learning rates for different behaviors and categorizations (α_B1,C1_ for the human opponent’s choice to compete; α_B1,C2_ for the computer opponent’s choice to compete; α_B2,C1_ for the human opponent’s choice to cooperate; and α_B2,C2_ for the computer opponent’s choice to cooperate). Model_2BPC considered the joint effect of all three levels of information and set different learning rates for each level of information (α_B1,P1,C1_ for the highly competitive human opponent’s choice to compete; α_B1,P2,C1_ for the less competitive human opponent’s choice to compete; α_B2,P1,C1_ for the highly competitive human opponent’s choice to cooperate; α_B2,P2,C1_ for the less competitive human opponent’s choice to cooperate; α_B1,P1,C2_ for the highly competitive computer opponent’s choice to compete; α_B1,P2,C2_ for the less competitive computer opponent’s choice to compete; α_B2,P1,C2_ for the highly competitive computer opponent’s choice to cooperate; and α_B2,P2,C2_ for the less competitive computer opponent’s choice to cooperate). The first three models above aim to examine the independent effects of each information level, while the others explore the synergistic impact of combining these levels.

The model fitting and parameter estimation process followed the methodology used in Experiment 1, as well as statistical analysis of behavioral data and model parameters (for more details, refer to the Supplementary Information).

#### 2.2.2. Result

- Behavioral results

**Figure 3:**
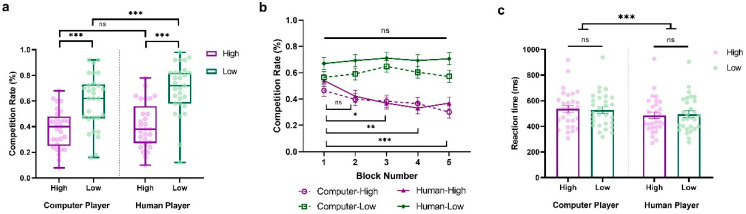
Results from Experiment 2. (**a**) Average percentage of decisions to competition (go straight), (**b**) block-to-block evolution of competition rate %, and (**c**) overall reaction time (ms) when participants play against highly and less competitive human and computer players. The *y*-axis indicates the raw performance data, with each dot representing an individual participant (* indicates *p* < 0.05, ** indicates *p* < 0.005, *** indicates *p* < 0.001, and ns indicates non-significant; error bars represent SEM).

We conducted a three-way repeated-measure ANOVA of the competition rate, with Sociality (Human vs. Computer), Competition (High vs. Low), and Block (Block1~Block5) as within-subject variables. The main effects of Sociality (*F*_(1,28)_ = 16.037, *p* < 0.001, η_p_^2^ = 0.364, BF_10_ = 18.636) and Competition (*F*_(1,28)_ = 31.392, *p* < 0.001, η_p_^2^ = 0.528, BF_10_ = 12,335.938) were significant. These results revealed that participants were more competitive against human opponents than computers, and against less competitive players compared to highly competitive ones (HCP: *M* = 0.382, *SD* = 0.153; LCP: *M* = 0.596, *SD* = 0.211; HHP: *M* = 0.405, *SD* = 0.178; LHP: *M* = 0.695, *SD* = 0.199). Interestingly, we also observed a significant interaction between Sociality and Competition (*F*_(1,28)_ = 4.395, *p* = 0.045, *η_p_*^2^ = 0.136, BF_10_ = 2.555). As shown in Figure 3a, when participants were facing a highly competitive player, they tended to behave similarly. However, when they were facing a less competitive player, they tended to be more competitive against a human player than a computer (HCP vs. HHP: *t* = −1.017, *p* = 1.000, Cohen’s *d* = −0.098, 95% CI = [−0.362, 0.166]; LCP vs. LHP: *t* = −4.199, *p* < 0.001, Cohen’s *d* = −0.405, 95% CI = [−0.705, −0.105]).

Moreover, we also observed a significant interaction between Competition and Block (*F*_(4,112)_ = 3.880, *p* = 0.005, *η_p_*^2^ = 0.122, BF_10_ = 7.408), along with the significant main effect of Block (*F*_(4,112)_ = 2.952, *p* = 0.023, *η_p_*^2^ = 0.095, BF_10_ = 2.605), suggesting that participants’ competitive behaviors evolved over time and varied according to the competitiveness of the opponent (see Figure 3b). Consistent with Experiment 1, participants exhibited stable competitive behaviors against less competitive players, with no notable differences across blocks (*p*s > 0.05). In contrast, against highly competitive players, their competitive behaviors progressively decreased over the blocks, with the differences between blocks becoming increasingly significant (Block 1 vs. Block 2: *t* = 2.496, *p* = 0.599, Cohen’s *d* = 0.384, 95% CI = [−0.158, 0.927]; Block 1 vs. Block 3: *t* = 3.365, *p* = 0.041, Cohen’s *d* = 0.518, 95% CI = [−0.046, 1.082]; Block 1 vs. Block 4: *t* = 4.097, *p* = 0.003, Cohen’s *d* = 0.631, 95% CI = [0.045, 1.218]; Block 1 vs. Block 5: *t* = 4.463, *p* < 0.001, Cohen’s *d* = 0.687, 95% CI = [0.089, 1.286]).

Further, we conducted a two-way repeated-measure ANOVA on reaction time with Sociality (Human vs. Computer) and Competition (High vs. Low) as within-subject variables. The findings showed a significant main effect of Sociality, indicating that participants responded more quickly when competing against human opponents than computers (*F*_(1,28)_ = 28.242, *p* < 0.001, *η_p_*^2^ = 0.502, BF_10_ = 335.364; HCP: *M* = 535.371, *SD* = 143.456; LCP: *M* = 526.935, *SD* = 136.171; HHP: *M* = 484.024, *SD* = 138.978; LHP: *M* = 494.745, *SD* = 138.365; See Figure 3c). No other significant main effects or interactions were observed (Competition: *F*_(1,28)_ = 0.017, *p* = 0.896, *η_p_*^2^ = 6.183 × 10^−4^, BF_10_ = 0.273; Sociality × Competition: *F*_(1,28)_ = 1.140, *p* = 0.295, *η_p_*^2^ = 0.039, BF_10_ = 0.568).

- Revelation in the computational model

Similar to what we observed in Experiment 1, the comparative analysis of the computational models in Experiment 2 revealed that Model_2B was more effective in fitting the participants’ data than other models (Model_2P: *t*_(28)_ = −2.30, *p* < 0.05, Cohen’s *d* = −0.43; Model_2C: *t*_(28)_ = −3.09, *p* < 0.01, Cohen’s *d* = −0.57; Model_2BP: *t*_(28)_ = −3.44, *p* < 0.01, Cohen’s *d* = −0.64; Model_2BC: *t*_(28)_ = −4.34, *p* < 0.001, Cohen’s *d* = −0.81; Model_2PC: *t*_(28)_ = −3.99, *p* < 0.001, Cohen’s *d* = −0.74; Model_2BPC: *t*_(28)_ = −6.02, *p* < 0.001, Cohen’s *d* = −1.12). Based on Model_2B, using a paired-sample *t*-test, we found that the learning rate when players chose cooperation was higher than that of players choosing competition (*t*_(28)_ = −2.201, *p* = 0.036, Cohen’s *d* = −0.409, 95% CI = [−0.785, −0.026], BF_10_ = 1.571; HP: *M* = 0.091, *SD* = 0.088; LP: *M* = 0.196, *SD* = 0.265). Detailed results of the model estimation and comparison are shown in Table 2.

### 2.3. Experiment 3

In Experiment 3, we retained the framework of behavior, pattern, and categorization information from Experiment 2 but introduced a more complex and uncertain environment. The focus was on increasing the intricacy of opponent profiles by presenting participants with a variety of human and computer opponents whose behavioral patterns were less predictable and more variable. In this experiment, we aim to further examine how the dynamics among behavior, pattern, and categorization impact our decision-making strategies in a context closer to our daily environment.

#### 2.3.1. Methods

- Participants

A priori sample size estimation was conducted using G. Power V3.1 with the following parameters: repeated-measure design factors = 2 × 2 × 3; effect size = 0.25; significance level = 0.05; and power = 0.9. The power analysis resulted in a sample of twenty-four participants. An independent group of twenty-nine healthy undergraduate participants (aged 20.50 ± 2.25 years, 16 females) were recruited through internet advertisements and provided informed written consent. All participants are right-handed, with no history of psychiatric or neurological disorder, and have normal or corrected normal vision.

- Experimental design and procedure

Experiment 3 replicated the Chicken Game structure from the first two experiments, with identical scoring rules and a mix of four human and computer opponents. The stimuli and trial illustration are identical to Experiments 1 and 2. However, in this experiment (see Figure 1), we introduced a new experimental design over 6 blocks, divided into three distinct phases. In Phase 1 (Block 1), all opponents were programmed to have an equal and neutral probability of choosing to go straight (50%). Phase 2 (Blocks 2 to 4) aligned with the structure of Experiment 2, categorizing opponents based on competitiveness—human and computer players with either high (70% probability) or low (30% probability) tendencies to go straight. The final phase, Phase 3 (Blocks 5 and 6), reverted to the initial setup of a 50% straight-going probability for all opponents. This design aimed to investigate how participants dynamically navigated decision-making amidst shifting levels of competitive behavior.

- Model Construction and Estimation

For Experiment 3, the construction and estimation of models followed the same methodology as the seven models we developed in Experiment 2 (see Supplementary Information).

#### 2.3.2. Result

- Behavioral results

**Figure 4:**
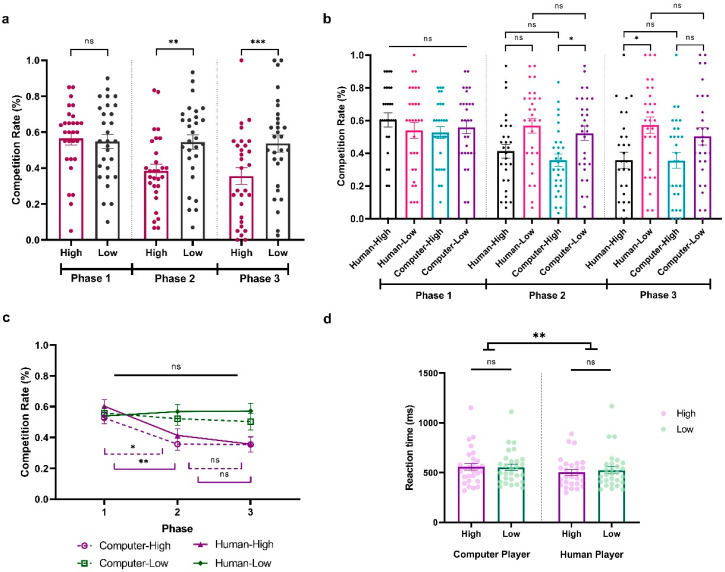
Results from Experiment 3. (**a**) Average percentage of decisions to competition (go straight) when facing highly and less competitive players across phases. (**b**) Average percentage of decisions for competition when facing highly and less competitive human and computer players across phases. (**c**) Time effect when participant is facing different players. (**d**) Average reaction time (ms) when participants play against highly and less competitive human and computer players. The *y*-axis indicates the raw performance data, with each dot representing an individual participant (* indicates *p* < 0.05, ** indicates *p* < 0.005, *** indicates *p* < 0.001, and ns indicates non-significant; error bars represent SEM).

To explore how participants adapted to the fluctuating competition levels of their opponents in Experiment 3, we conducted a three-way repeated ANOVA on participants’ competition rate, with Sociality (Human vs. Computer), Competition (High vs. Low), and Phase (Phase 1, Phase 2, Phase 3) as within-subject variables. The results revealed significant main effects of Competition (*F*_(1,28)_ = 15.649, *p* < 0.001, *η_p_*^2^ = 0.359, BF_10_ = 4023.234) and Phase (*F*_(2,56)_ = 6.948, *p* < 0.01, *η_p_*^2^ = 0.199, BF_10_ = 1149.606) and a significant interaction between them (*F*_(2,56)_ = 9.882, *p* < 0.001, *η_p_*^2^ = 0.261, BF_10_ = 311.991). There was also a significant three-way interaction among Sociality, Competition, and Phase (*F*_(2,56)_ = 3.248, *p* = 0.046, *η_p_*^2^ = 0.104, BF_10_ = 0.547). To better understand the effect, we first look at each phase separately. During Phase 1, with all opponents at a neutral competition level, participants showed no significant competitive behavior differences (All *p*s > 0.05; HCP: *M* = 0.526, *SD* = 0.198; LCP: *M* = 0.558, *SD* = 0.215; HHP: *M* = 0.603, *SD* = 0.231; LHP: *M* = 0.539, *SD* = 0.264; Figure 4a,b). Contrastingly, in Phase 2, their competitiveness increased against less competitive players (*t* = 4.084, *p* = 0.002, Cohen’s *d* = −0.660, 95% CI = [−1.218, −0.102], Figure 4a). Moreover, this adaptation behavior seems stronger when they are facing computer players, with a significantly higher competition level in less competitive computer behavior compared to highly competitive behavior (HCP vs. LCP: (*t* = −3.562, *p* = 0.034, Cohen’s *d* = −0.683, 95% CI = [−1.419, 0.054]; HCP: *M* = 0.356, *SD* = 0.204; LCP: *M* = 0.522, *SD* = 0.238; Figure 4b). However, this was not the case when they were facing the human players (HHP vs. LHP: *t* = −3.327, *p* = 0.076, Cohen’s *d* = −0.638, 95% CI = [−1.365, −0.090]; HHP: *M* = 0.413, *SD* = 0.226; LHP: *M* = 0.568, *SD* = 0.244; Figure 4b). Remarkably, in Phase 3, when all opponents returned to neutral competition rates, in general, participants still showed a significant difference in competition behavior when they were facing a less compared to highly competitive player (*t* = 4.084, *p* < 0.001, Cohen’s *d* = −0.745, 95% CI = [−1.320, −0.169]; Figure 4a). This pattern was reversed when we looked at human and computer players separately. Specifically, participants showed a continued increased difference in competition rate when they were facing the human players (HHP vs. LHP: *t* = −4.586, *p* < 0.001, Cohen’s *d* = −0.879, 95% CI = [−1.659, −0.099]; HHP: *M* = 0.356, *SD* = 0.270; LHP: *M* = 0.571, *SD* = 0.271) but not the computer players (HCP vs. LCP: *t* = −3.184, *p* = 0.120, Cohen’s *d* = −0.610, 95% CI = [−1.333, 0.113]; HCP: *M* = 0.354, *SD* = 0.257; LCP: *M* = 0.503, *SD* = 0.289; Figure 4b).

Next, an additional analysis focused on the Phase effect of participants’ competitive behaviors when they were facing different players. As shown in Figure 4c, although the competition level of the less competitive computer player significantly varied from Phase 1 to Phase 3, participants did not significantly alter their competitive responses across these phases (LCP; Phase 1 vs. Phase 2: *t* = 0.805, *p* = 1.000, Cohen’s *d* = 0.149, 95% CI = [−0.495, 0.793]; Phase 2 vs. Phase 3: *t* = 0.427, *p* = 1.000, Cohen’s *d* = 0.079, 95% CI = [−0.563, 0.720]). This was the same when they were facing the less competitive human player (LHP; Phase 1 vs. Phase 2: *t* = −0.652, *p* = 1.000, Cohen’s *d* = −0.120, 95% CI = [−0.763, −0.522]; Phase 2 vs. Phase 3: *t* = −0.059, *p* = 1.000, Cohen’s *d* = −0.011, 95% CI = [−0.651, 0.630]). Notably, when participants were facing both highly competitive humans and competitive behavior, they demonstrated an adaptive response in Phase 2 by reducing their competitive behaviors (HCP Phase 1 vs. Phase 2: *t* = 3.789, *p* = 0.014, Cohen’s *d* = 0.699, 95% CI = [−0.019, 1.417]; HHP Phase 1 vs. Phase 2: *t* = 4.241, *p* = 0.003, Cohen’s *d* = 0.783, 95% CI = [0.047, 1.519]). However, this sensitivity was not as pronounced in Phase 3 when both highly competitive human and computer players lowered their competition rates, as evidenced by the lack of a significant behavioral change (HCP Phase 2 vs. Phase 3: *t* = 0.033, *p* = 1.000, Cohen’s *d* = 0.006, 95% CI = [−0.634, 0.647]; HHP Phase 2 vs. Phase 3: *t* = 1.249, *p* = 1.000, Cohen’s *d* = 0.231, 95% CI = [−0.419, 0.880]). In conclusion, our findings indicate that participants’ adaptive responses to competitive behaviors are nuanced and influenced by both the intensity of the competition and the specific phase in which the changes take place.

We further conducted a two-way repeated-measure ANOVA on reaction time with Sociality (Human vs. Computer) and Competition (High vs. Low) as within-subject variables. The results showed a significant main effect of Sociality, indicating that participants exhibited faster reaction times when competing against human opponents compared to computers (*F*_(1,28)_ = 7.575, *p* = 0.010, *η_p_*^2^ = 0.213, BF_10_ = 4.607; HCP: *M* = 556.759, *SD* = 179.252; LCP: *M* = 552.986, *SD* = 167.567; HHP: *M* = 505.216, *SD* = 146.394; LHP: *M* = 527.563, *SD* = 182.042; see Figure 4d). No additional significant main effects or interactions were observed (Competition: *F*_(1,28)_ = 1.764, *p* = 0.195, *η_p_*^2^ = 0.059, BF_10_ = 0.472; Sociality × Competition: *F*_(1,28)_ = 2.472, *p* = 0.127, *η_p_*^2^ = 0.081, BF_10_ = 0.889).

- Revelation in the computational model

The comparative analysis of the computational models in Experiment 3 demonstrated that Model_3P provided a significantly better fit to the participants’ data compared to other models (Model_3B: *t*_(28)_ = −4.19, *p* < 0.001, Cohen’s *d* = −0.78; Model_3C: *t*_(28)_ = −2.80, *p* < 0.01, Cohen’s *d* = −0.52; Model_3BP: *t*_(28)_ = −25.16, *p* < 0.001, Cohen’s *d* = −4.67; Model_3BC: *t*_(28)_ = −11.17, *p* < 0.001, Cohen’s *d* = −2.07; Model_3PC: *t*_(28)_ = −12.79, *p* < 0.001, Cohen’s *d* = −2.37; Model_2BPC: *t*_(28)_ = −46.57, *p* < 0.001, Cohen’s *d* = −8.65). Additionally, based on Model_3P, a paired-sample *t*-test revealed that participants exhibited a significantly higher learning rate when playing with highly competitive players compared to less competitive players (*t*_(28)_ = 2.751, *p* = 0.010, Cohen’s *d* = 0.511, 95% CI = [0.119, 0.894], BF_10_ = 4.440; HP: *M* = 0.130, *SD* = 0.222; LP: *M* = 0.057, *SD* = 0.185). These findings highlight the distinct learning dynamics exhibited by participants in response to varying levels of opponent competitiveness, with a pronounced preference for adapting to more challenging interactions. Detailed results of the model estimation and comparison are shown in Table 3.

## 3. Discussion

Competition or cooperation presents a common interpersonal dilemma where, driven by the greater interest, individuals tend to compete, but the risk of significant losses for both sides may lead them to choose cooperation. However, effective cooperation requires alignment of intentions from all parties involved [34]. Acquiring information about the other party becomes crucial in guiding our decisions. However, objective information may not be entirely retained after subjective processing, leading to loss or distortion at three levels of information, i.e., behavior, pattern, and categorization. Through three experiments and reinforcement learning models, our study examined the impact of different information levels on decision-making. We observed that participants adapted their competitiveness in response to their opponents’ behaviors, showing less competitiveness against highly competitive players and more against those who are less competitive (across three experiments). This adaptation rate varied depending on the opponents’ competitiveness: participants gradually decreased their competitiveness when confronted with highly competitive players, whereas they rapidly increased their competitiveness when confronted with less competitive players. This behavioral bias may arise at the level of behavior in the hierarchical framework of information acquisition, where participants showed a lower learning rate for competitive behavior compared to cooperation behavior (Experiment 1 and Experiment 2). Furthermore, in scenarios with constantly changing patterns of opponents, participants were more reactive to competitors who previously exhibited high competitiveness, emphasizing the significance of pattern-level information in decision-making (Experiment 3).

### 3.1. Complementary Behavioral Patterns and Asymmetric Adaptations

The findings from our three experiments indicate that participants adapt their behavior in response to that of their opponents, demonstrating complementary behavioral characteristics. The Chicken Game is a classic anti-coordination game where both sides competing leads to the greatest loss for each player, while choosing to compete when others choose to cooperate yields the highest benefit [18,35]. In this type of game, there is no optimal choice; the best strategy is to make the exact opposite choice of the opponent [5,18,36,37]. Under such scoring rules, the complementary behavioral patterns between participants and opponents were reasonable, meaning that participants showed decreased competitiveness against highly competitive opponents and increased competitiveness against less competitive ones. This strategy, aiming to maximize gains and minimize losses in the anti-coordination game, aligns with previous studies.

For instance, using the Chicken Game, one study explored the effect of opponents’ behavioral tendencies on interpersonal cooperation [38]. Participants were randomly assigned to play with either a competitive opponent (20% cooperative rate) or a cooperative opponent (80% cooperative rate). The results indicated that participants became more cooperative when paired with competitive opponents and more competitive with cooperative opponents. In our study, using 70% and 30% cooperative rates, we observed similar complementary behavioral patterns. Together, these results emphasize the significance of opponent behavior tendencies in influencing individual decision-making in competitive and cooperative dilemmas.

However, the more interesting findings from our study lie in the bias in adaptation rates to competitive versus cooperative opponents. Specifically, our results demonstrate that participants adapted more slowly to the highly competitive opponent than to the cooperative one. This asymmetry behavioral performance provides deeper insight into the strategic decision-making processes and highlights the nuanced ways in which individuals adjust their behavior based on the perceived competitiveness of their opponents.

### 3.2. The Effect of “Behavior” Level on Asymmetric Adaptation

The complementary behavioral characteristic we observed in our study illustrated how individuals learn and adapt their behavior based on their opponents’ behaviors in dynamic interactions of cooperation and competition. This adaptation is part of a broader process of social learning, where individuals continuously gather and update information about others’ behaviors. In both Experiment 1 and Experiment 2, set within the framework of the Chicken Game, participants encountered opponents with stable behavioral patterns. We observed that participants adapted at varying rates depending on their opponents’ level of competitiveness: they gradually reduced their competitiveness over five blocks against highly competitive opponents, whereas they swiftly increased their competitiveness in just one block when facing less competitive opponents.

The observed asymmetric behavioral adaptation in our study might be attributed to different levels of information processing, including behavior, pattern, and categorization. To delve into this phenomenon, we constructed reinforcement learning models that evaluated the influence of each level and their interactions. Our results indicated that the model that differentiated opponents’ behavior choices aligned closely with participants’ actual behavior. Additionally, participants showed a higher learning rate when receiving feedback on cooperation compared to feedback on competition. Participants were quicker to learn from cooperative feedback than from competitive feedback, enabling them to swiftly adapt their strategies against less competitive, more cooperative opponents, thereby enhancing their competitiveness and obtaining higher scores. In contrast, the slower acquisition of competitive behaviors resulted in a more gradual reduction in competitiveness against highly competitive opponents. This asymmetric learning pattern of binary behavior (or feedback) is also evident in other contexts. Studies have shown that learning about gains and losses is asymmetric, with distinct neural underpinnings that support adaptive learning from positive and negative outcomes [39,40]. Research on social appraisal linked personality traits with differences in learning from social feedback (approval and disapproval feedback) and found that individuals with low self-esteem showed a slower learning rate from positive feedback compared to those with high self-esteem [41]. Furthermore, an fMRI study examined brain activity in regions associated with the theory of mind during cooperative and competitive interactions with the same individual within the same paradigm. It found that these brain regions encoded differences between cooperation and competition [42]. These findings collectively suggest that the observed biases in social adaptation to competitive behavior, particularly in contexts of competition and cooperation dilemmas, are part of a broader pattern of asymmetric information processing and learning.

### 3.3. The Effect of “Pattern” Level on Asymmetric Adaptation

Contrasting with the settings of Experiment 1 and Experiment 2, Experiment 3 presented participants with a context characterized by fluctuating competition levels from opponents. The influence of uncertainty in competition and cooperation scenarios is well documented [5,43]. In this context, we observed that participants were particularly responsive to opponents who displayed high levels of competitiveness, adapting their behavior to match these opponents. When opponents did not consistently exhibit high competitiveness, participants tended to respond with a neutral level of competitiveness.

This characteristic is also captured by the computational model. The validation of various levels of information influencing decision-making suggests that asymmetric learning of behavioral patterns may be a potential explanation for participants’ performance in fluctuating contexts. High competitiveness from an opponent, especially in uncertain situations, can be perceived as a dominant or threatening signal, prompting individuals to maintain vigilant attention and update their strategies accordingly [44,45,46]. In Experiment 1 and Experiment 2, with stable opponent behavior patterns, participants could learn and anticipate these patterns, appropriately selecting competitive actions to optimize their scores. Thus, close attention to the opponent’s behavioral choices in each trial was more crucial in these stable contexts. However, in fluctuating situations as seen in Experiment 3, recognizing opponents’ behavior patterns becomes a challenging task, requiring ongoing adjustment and evaluation of these patterns. This finding aligns with a previous study that also demonstrated the effect of predictable levels of opponent behavior patterns on individuals’ use of strategies in competitive interactions. Unpredictable behavior patterns make it difficult for interactors to effectively optimize their own decisions, rendering the opponent unexploitable. When participants encounter different styles of opponents, such as unexploitable and exploitable opponents, within the same experimental session, random behavior can be observed against unexploitable opponents [47]. These findings highlight the profound impact of social uncertainty on the decision-making process, particularly from the early stages of acquiring information about others’ behavior patterns.

### 3.4. The Effect of “Categorization” Level on Asymmetric Adaptation

How does social categorization, specifically whether participants perceive their opponent as human or computer, affect asymmetric adaptation? Our findings reveal a consistent main effect of Sociality across Experiments 2 and 3, where participants responded more quickly and competitively when interacting against human opponents than against computers. The heightened competitiveness may be driven by an intrinsic human motivation to compete for status, dominance, and social rewards, which is more pronounced when the opponent is human [48,49]. This suggests that categorizing an opponent as human activates deeper social and evolutionary drives to compete, which is less pronounced when the opponent is a non-human entity such as a computer.

Participants’ faster reaction times when competing against human opponents suggest that they were more engaged or motivated in these trials, likely due to the increased social and cognitive demands associated with human interactions. This finding aligns with the theory of social facilitation, which posits that the presence of others, particularly in competitive scenarios, can enhance individual performance [50]. Additionally, humans are more likely to engage in complex cognitive processes, such as the Theory of Mind, when competing against other humans, anticipating their actions, and reacting accordingly [51]. Conversely, one plausible explanation for the slower reaction times observed with computer opponents is that participants may engage in more deliberate and cautious decision-making [52]. Given that they are interacting with a predictable algorithm, participants might take additional time to strategize and anticipate the computer’s next move, resulting in more calculated, but slower, responses [53,54]. In contrast, the inherent unpredictability and variability of human behavior may lead participants to rely more on intuitive and heuristic-based responses, contributing to quicker reaction times in competitive human scenarios [55].

The differential response times between human and computer opponents underscore the influence of social categorization on behavior, suggesting that participants may have perceived human opponents as more challenging or worthy competitors, thereby increasing their arousal and performance, evidenced by a higher competitive rate and faster reaction time in our studies. Together, these findings suggest that the social categorization of an opponent plays a critical role in modulating cognitive and behavioral responses during competition and cooperation dilemmas. This has broader implications for understanding how the perceived social presence and human-like characteristics of opponents can influence performance in both virtual and real-world competitive environments.

### 3.5. Limitations and Future Directions

While our study provides valuable insights into how individuals navigate competitive and cooperative interactions, we acknowledge that the limitations of our sample and the controlled laboratory setting of the Chicken Game may not fully capture the complexity of real-world dynamics. The use of deception, where participants believed they were playing against humans, may compromise the ecological validity of this study.

Given these limitations, future research should aim to include larger and more diverse samples to enhance generalizability. Investigating these dynamics in more naturalistic settings or through field experiments could help validate findings in real-world situations. Additionally, exploring a broader range of probabilities (i.e., 55%, 65%, and 75%) to determine the threshold at which differences in competitive behavior become significant could provide a more nuanced understanding of how subtle changes in opponent behavior affect participants’ strategies. Moreover, employing different types of games and testing models could provide a more comprehensive understanding of these dynamics. Last but not least, one interesting finding from our study is the difference in reaction times when participants faced human versus computer opponents, which could contribute to our understanding of human–robot interactions. Incorporating neuroimaging or psychophysiological measures in future studies could reveal the underlying neural and psychological mechanisms driving the observed behaviors and adaptation rates.

## 4. Conclusions

In conclusion, our current study investigated the dynamics of social learning in competition–cooperation dilemmas. Across three experiments, participants engaged in the Chicken Game, encountering opponents with varying degrees of competitiveness, along with distinct behavioral patterns and categorizations. Employing computational modeling, we decoded the social learning process, focusing on the acquisition of information across a behavior-pattern-categorization hierarchy. Overall, our findings reveal that individuals exhibited different rates of social adaptation to opponents with different levels of competitiveness and fluctuations. More importantly, we observed that individuals exhibited different rates of social adaptation to opponents with different levels of competitiveness and fluctuations. This asymmetric adaptation in behavior is closely tied to the rate at which different levels of information are updated during social learning. The current study sheds light on how individuals navigate competition and cooperation dilemmas, highlighting the role of social information processing at various levels in shaping decision-making strategies.

## Figures and Tables

**Table 1:** Means (standard error mean) of model parameters in Experiment 1.

Information Level	Parameter	M_1B	M_1P	M_1BP
	β	0.23 (0.04)	0.23 (0.03)	0.50 (0.002)
	AIC	63.05 (1.52)	65.05 (1.61)	66.55 (1.55)
Behavior	α_B1_	0.06 (0.01)		
	α_B2_	0.21 (0.07)		
Pattern	α_P1_		0.09 (0.04)	
	α_P2_		0.06 (0.02)	
Behavior + Pattern	α_B1,P1_			0.10 (0.01)
	α_B1,P2_			0.07 (0.02)
	α_B2,P1_			0.21 (0.05)
	α_B2,P2_			0.26 (0.07)

Subscripts of α: B, behavior information; B1, competition; B2, cooperation; P, pattern information; P1, highly competitive; P2, less competitive.

**Table 2:** Means (standard error mean) of model parameters in Experiment 2.

Information Level	Parameter	M_2B	M_2P	M_2C	M_2BP	M_2BC	M_2PC	M_2BPC
	β	0.21 (0.05)	0.21 (0.04)	0.20 (0.04)	0.26 (0.05)	0.21 (0.04)	0.22 (0.04)	0.22 (0.04)
	AIC	121.58 (3.80)	125.14 (3.65)	128.18 (3.47)	124.01 (3.67)	123.50 (3.70)	127.93 (3.67)	127.94 (3.77)
Behavior	α_B1_	0.09 (0.02)						
	α_B2_	0.20 (0.05)						
Pattern	α_P1_		0.05 (0.01)					
	α_P2_		0.11 (0.02)					
Categorization	α_C1_			0.25 (0.06)				
	α_C2_			0.11 (0.03)				
Behavior + Pattern	α_B1,P1_				0.18 (0.05)			
	α_B1,P2_				0.12 (0.05)			
	α_B2,P1_				0.17 (0.05)			
	α_B2,P2_				0.17 (0.05)			
Behavior + Categorization	α_B1,C1_					0.10 (0.02)		
	α_B1,C2_					0.14 (0.04)		
	α_B2,C1_					0.23 (0.05)		
	α_B2,C2_					0.13 (0.04)		
Pattern + Categorization	α_P1,C1_						0.05 (0.01)	
	α_P1,C2_						0.12 (0.03)	
	α_P2,C1_						0.05 (0.01)	
	α_P2,C2_						0.09 (0.02)	
Behavior + Pattern + Categorization	α_B1,P1,C1_							0.13 (0.04)
	α_B1,P1,C2_							0.11 (0.04)
	α_B1,P2,C1_							0.33 (0.07)
	α_B1,P2,C2_							0.11 (0.02)
	α_B2,P1,C1_							0.16 (0.04)
	α_B2,P1,C2_							0.13 (0.04)
	α_B2,P2,C1_							0.08 (0.02)
	α_B2,P2,C2_							0.20 (0.06)

Subscripts of α: B, behavior information; B1, competition; B2, cooperation; P, pattern information; P1, highly competitive; P2, less competitive; C, categorization information; C1, social; C2, non-social.

**Table 3:** Means (standard error mean) of model parameters in Experiment 3.

Information Level	Parameter	M_3B	M_3P	M_3C	M_3BP	M_3BC	M_3PC	M_3BPC
	β	0.05 (0.01)	0.05 (0.01)	0.05 (0.01)	0.05 (0.01)	0.05 (0.01)	0.05 (0.01)	0.05 (0.01)
	AIC	159.58 (5.08)	157.41 (5.24)	158.72 (5.15)	161.69 (5.26)	162.66 (5.14)	161.22 (5.29)	168.80 (5.25)
Behavior	α_B1_	0.04 (0.01)						
	α_B2_	0.54 (0.02)						
Pattern	α_P1_		0.13 (0.05)					
	α_P2_		0.06 (0.03)					
Categorization	α_C1_			0.16 (0.06)				
	α_C2_			0.10 (0.04)				
Behavior + Pattern	α_B1,P1_				0.11 (0.03)			
	α_B1,P2_				0.12 (0.05)			
	α_B2,P1_				0.51 (0.01)			
	α_B2,P2_				0.52 (0.01)			
Behavior + Categorization	α_B1,C1_					0.10 (0.04)		
	α_B1,C2_					0.12 (0.05)		
	α_B2,C1_					0.51 (0.01)		
	α_B2,C2_					0.51 (0.01)		
Pattern + Categorization	α_P1,C1_						0.14 (0.05)	
	α_P1,C2_						0.20 (0.06)	
	α_P2,C1_						0.30 (0.08)	
	α_P2,C2_						0.12 (0.05)	
Behavior + Pattern + Categorization	α_B1,P1,C1_							0.20 (0.06)
	α_B1,P1,C2_							0.17 (0.05)
	α_B1,P2,C1_							0.51 (0.02)
	α_B1,P2,C2_							0.03 (0.01)
	α_B2,P1,C1_							0.53 (0.02)
	α_B2,P1,C2_							0.49 (0.01)
	α_B2,P2,C1_							0.26 (0.07)
	α_B2,P2,C2_							0.48 (0.02)

Subscripts of α: B, behavior information; B1, competition; B2, cooperation; P, pattern information; P1, highly competitive; P2, less competitive; C, categorization information; C1, social; C2, non-social.

## Data Availability

The data that support the findings of this study are available from the corresponding author upon reasonable request.

## Supplementary Materials

The following supporting information can be downloaded at: https://www.mdpi.com/article/10.3390/bs14080721/s1, Experiments 1–3.
