# Supplementary material for: Asymmetric Adaption in Social Learning: Understanding the Dilemma of Competition and Cooperation
Source: Behav Sci (Basel). 2024 Aug 16;14(8):721. doi: 10.3390/bs14080721 (PMC11351303; doi:10.3390/bs14080721)
Supplement: Supplementary file 1 [file behavsci-14-00721-s001.zip › behavsci-3126819-supplementary.pdf]

# Supplementary Information

## Experiment 1

Model_1B

$$P(d)_{t+1} = P(d)_t + \alpha_B * PE_t \quad (1)$$

$$PE_t = \gamma_t - P(d)_t \quad (2)$$

$$EV(d)_t = P(d)_t * (-30) + (1 - P(d)_t) * 30 \quad (3)$$

$$EV(s)_t = P(d)_t * (-10) + (1 - P(d)_t) * 10 \quad (4)$$

$$IP(d)_t = \frac{e^{\frac{EV(d)_t}{\beta}}}{e^{\frac{EV(d)_t}{\beta}} + e^{\frac{EV(s)_t}{\beta}}} \quad (5)$$

$$LLE = \sum_{t=1}^{n} \log(IP_t) \quad (6)$$

Free parameters:
α = learning rate; β = temperature
Index:
t = the number of trial; B = Behavior

Model_1P

$$P(d)_{t+1} = P(d)_t + \alpha_P * PE_t \quad (1)$$

$$PE_t = \gamma_t - P(d)_t \quad (2)$$

$$EV(d)_t = P(d)_t * (-30) + (1 - P(d)_t) * 30 \quad (3)$$

$$EV(s)_t = P(d)_t * (-10) + (1 - P(d)_t) * 10 \quad (4)$$

$$IP(d)_t = \frac{e^{\frac{EV(d)_t}{\beta}}}{e^{\frac{EV(d)_t}{\beta}} + e^{\frac{EV(s)_t}{\beta}}} \quad (5)$$

$$LLE = \sum_{t=1}^{n} \log(IP_t) \quad (6)$$

Free parameters:
α = learning rate; β = temperature
Index:
t = the number of trial; P = Pattern

Model_1BP

$$P(d)_{t+1} = P(d)_t + \alpha_{B,P} * PE_t \quad (1)$$

$$PE_t = \gamma_t - P(d)_t \quad (2)$$

$$EV(d)_t = P(d)_t * (-30) + (1 - P(d)_t) * 30 \quad (3)$$

$$EV(s)_t = P(d)_t * (-10) + (1 - P(d)_t) * 10 \quad (4)$$

$$IP(d)_t = \frac{e^{\frac{EV(d)_t}{\beta}}}{e^{\frac{EV(d)_t}{\beta}} + e^{\frac{EV(s)_t}{\beta}}} \quad (5)$$

$$LLE = \sum_{t=1}^{n} \log(IP_t) \quad (6)$$

Free parameters:
α = learning rate; β = temperature
Index:
t = the number of trial; B = Behavior; P = Pattern

## Experiment 2&3

Model_2B&Model_3B

$$P(d)_{t+1} = P(d)_t + \alpha_B * PE_t \quad (1)$$

$$PE_t = \gamma_t - P(d)_t \quad (2)$$

$$EV(d)_t = P(d)_t * (-30) + (1 - P(d)_t) * 30 \quad (3)$$

$$EV(s)_t = P(d)_t * (-10) + (1 - P(d)_t) * 10 \quad (4)$$

$$IP(d)_t = \frac{e^{\frac{EV(d)_t}{\beta}}}{e^{\frac{EV(d)_t}{\beta}} + e^{\frac{EV(s)_t}{\beta}}} \quad (5)$$

$$LLE = \sum_{t=1}^{n} \log(IP_t) \quad (6)$$

Free parameters:
α = learning rate; β = temperature
Index:
t = the number of trial; B = Behavior

Model_2P&Model_3P

$$P(d)_{t+1} = P(d)_t + \alpha_P * PE_t \quad (1)$$

$$PE_t = \gamma_t - P(d)_t \quad (2)$$

$$EV(d)_t = P(d)_t * (-30) + (1 - P(d)_t) * 30 \quad (3)$$

$$EV(s)_t = P(d)_t * (-10) + (1 - P(d)_t) * 10 \quad (4)$$

$$IP(d)_t = \frac{e^{\frac{EV(d)_t}{\beta}}}{e^{\frac{EV(d)_t}{\beta}} + e^{\frac{EV(s)_t}{\beta}}} \quad (5)$$

$$LLE = \sum_{t=1}^{n} \log(IP_t) \quad (6)$$

Free parameters:
α = learning rate; β = temperature
Index:
t = the number of trial; P = Pattern

Model_2C& Model_3C

$$P(d)_{t+1} = P(d)_t + \alpha_C * PE_t \quad (1)$$

$$PE_t = \gamma_t - P(d)_t \quad (2)$$

$$EV(d)_t = P(d)_t * (-30) + (1 - P(d)_t) * 30 \quad (3)$$

$$EV(s)_t = P(d)_t * (-10) + (1 - P(d)_t) * 10 \quad (4)$$

$$IP(d)_t = \frac{e^{\frac{EV(d)_t}{\beta}}}{e^{\frac{EV(d)_t}{\beta}} + e^{\frac{EV(s)_t}{\beta}}} \quad (5)$$

$$LLE = \sum_{t=1}^{n} \log(IP_t) \quad (6)$$

Free parameters:
α = learning rate; β = temperature
Index:
t = the number of trial; C = Categorization

Model_2BP& Model_3BP

$$P(d)_{t+1} = P(d)_t + \alpha_{B,P} * PE_t \quad (1)$$

$$PE_t = \gamma_t - P(d)_t \quad (2)$$

$$EV(d)_t = P(d)_t * (-30) + (1 - P(d)_t) * 30 \quad (3)$$

$$EV(s)_t = P(d)_t * (-10) + (1 - P(d)_t) * 10 \quad (4)$$

$$IP(d)_t = \frac{e^{\frac{EV(d)_t}{\beta}}}{e^{\frac{EV(d)_t}{\beta}} + e^{\frac{EV(s)_t}{\beta}}} \quad (5)$$

$$LLE = \sum_{t=1}^{n} \log(IP_t) \quad (6)$$

Free parameters:

α = learning rate; β = temperature

Index:

t = the number of trial; B = Behavior; P = Pattern

Model_2BC& Model_3BC

$$P(d)_{t+1} = P(d)_t + \alpha_{B,C} * PE_t \quad (1)$$

$$PE_t = \gamma_t - P(d)_t \quad (2)$$

$$EV(d)_t = P(d)_t * (-30) + (1 - P(d)_t) * 30 \quad (3)$$

$$EV(s)_t = P(d)_t * (-10) + (1 - P(d)_t) * 10 \quad (4)$$

$$IP(d)_t = \frac{e^{\frac{EV(d)_t}{\beta}}}{e^{\frac{EV(d)_t}{\beta}} + e^{\frac{EV(s)_t}{\beta}}} \quad (5)$$

$$LLE = \sum_{t=1}^{n} \log(IP_t) \quad (6)$$

Free parameters:

α = learning rate; β = temperature

Index:

t = the number of trial; B = Behavior; C = Categorization

Model_2PC& Model_3PC

$$P(d)_{t+1} = P(d)_t + \alpha_{P,C} * PE_t \quad (1)$$

$$PE_t = \gamma_t - P(d)_t \quad (2)$$

$$EV(d)_t = P(d)_t * (-30) + (1 - P(d)_t) * 30 \quad (3)$$

$$EV(s)_t = P(d)_t * (-10) + (1 - P(d)_t) * 10 \quad (4)$$

$$IP(d)_t = \frac{e^{\frac{EV(d)_t}{\beta}}}{e^{\frac{EV(d)_t}{\beta}} + e^{\frac{EV(s)_t}{\beta}}} \quad (5)$$

$$LLE = \sum_{t=1}^{n} \log(IP_t) \quad (6)$$

Free parameters:

α = learning rate; β = temperature

Index:

t = the number of trial; P = Pattern; C = Categorization

Model_2BPC& Model_3BPC

$$P(d)_{t+1} = P(d)_t + \alpha_{B,P,C} * PE_t \quad (1)$$

$$PE_t = \gamma_t - P(d)_t \quad (2)$$

$$EV(d)_t = P(d)_t * (-30) + (1 - P(d)_t) * 30 \quad (3)$$

$$EV(s)_t = P(d)_t * (-10) + (1 - P(d)_t) * 10 \quad (4)$$

$$IP(d)_t = \frac{e^{\frac{EV(d)_t}{\beta}}}{e^{\frac{EV(d)_t}{\beta}} + e^{\frac{EV(s)_t}{\beta}}} \quad (5)$$

$$LLE = \sum_{t=1}^{n} \log\left(IP_t\right) \quad (6)$$

Free parameters:

α = learning rate; β = temperature

Index:

t = the number of trial; B = Behavior; P = Pattern; C = Categorization
